# Developing longitudinal qualitative designs: lessons learned and recommendations for health services research

**DOI:** 10.1186/1471-2288-13-14

**Published:** 2013-02-06

**Authors:** Lynn Calman, Lisa Brunton, Alex Molassiotis

**Affiliations:** 1 University of Manchester, Jean McFarlane Building, Oxford Road, Manchester, M13 9PL, UK; 2 Hong Kong Polytechnic University, Hung Hom, Hong Kong

**Keywords:** Cancer, Health care, Users’ experiences, Interviews, Longitudinal studies, Research, Qualitative, Research design, Serial interview

## Abstract

**Background:**

Longitudinal qualitative methods are becoming increasingly used in the health service research, but the method and challenges particular to health care settings are not well described in the literature.We reflect on the strategies used in a longitudinal qualitative study to explore the experience of symptoms in cancer patients and their carers, following participants from diagnosis for twelve months; we highlight ethical, practical, theoretical and methodological issues that need to be considered and addressed from the outset of a longitudinal qualitative study.

**Results:**

Key considerations in undertaking longitudinal qualitative projects in health research, include the use of theory, utilizing multiple methods of analysis and giving consideration to the practical and ethical issues at an early stage. These can include issues of time and timing; data collection processes; changing the topic guide over time; recruitment considerations; retention of staff; issues around confidentiality; effects of project on staff and patients, and analyzing data within and across time.

**Conclusions:**

As longitudinal qualitative methods are becoming increasingly used in health services research, the methodological and practical challenges particular to health care settings need more robust approaches and conceptual improvement. We provide recommendations for the use of such designs. We have a particular focus on cancer patients, so this paper will have particular relevance for researchers interested in chronic and life limiting conditions.

## 

Longitudinal qualitative research (LQR) has been an emerging methodology over the last decade with methodological discussion and debate taking place within social research [1]. Longitudinal qualitative research is distinguished from other qualitative approaches by the way in which time is designed into the research process, making change a key focus for analysis [1]. LQR answers qualitative questions about the lived experience of change, or sometimes stability, over time. Findings can establish the processes by which this experience is created and illuminates the causes and consequences of change. Qualitative research is about why and how health care is experienced and LQR focuses on how and why these experiences change over time. In contrast to longitudinal quantitative methodologies LQR focuses on individual narratives and trajectories and can capture critical moments and processes involved in change. LQR is also particularly helpful in capturing “transitions” in care; for example, while researchers are beginning to more clearly map the cancer journey or pathway [2] we less clearly understand the processes involved in the experience of transition along this pathway whether that be to long term survivor or living with active or advanced disease. Saldana [3] identifies the principles that underpin LQR as duration, time and change and emphasizes that time and change are contextual and may transform during the course of a study.

Holland [4] identifies four methodological models of LQR.

• Mixed methods approaches. LQR may be imbedded within case studies, ethnographies and within quantitative longitudinal studies such as cohort studies and randomized controlled trials. Mixed methods studies are the context of most LQR studies in healthcare [5].

• Planned prospective longitudinal studies. Where the analysis can be the individual or the family or an organization.

• Follow-up studies, where an original study of participants are followed up after a period of time.

• Evaluation studies, for policy evaluation.

LQR methodologies can be particularly useful in assessing interventions. LQR studies embedded within randomized controlled trials or evaluation studies, of often complex interventions, are used as part of process evaluation. This can help us to understand not just whether an intervention may work but the mechanisms through which it works and if it is feasible and acceptable to the population under study [6].

LQR is becoming more frequently used in health research. LQR has been used, for example, to explore the prospect of dying [7], journeys to the diagnosis of cancer [8] and living with haemodialysis [9]. Published papers report mainly interview based studies, sometimes called serial interviews [10,11] to explore change over time, although other data collection methods are used. Different approaches have been taken to collection and analysis of data, for example, the use of longitudinal data to fully develop theoretical saturation of a category in a grounded theory study [12,13]. Data is not presented as a longitudinal narrative but as contributing to the properties of a category.

There are limitations in the published literature. Analysis is complex and multidimensional and can be tackled both cross-sectionally at each time point to allow analysis between individuals at the same time as well as longitudinally capturing each individual’s narrative. Thematic analysis is widely used [13-15] but can lead to cross-sectional descriptive accounts (what is happening at this time point) rather than focusing on causes and consequences of change. Research founded on explicit theoretical perspectives can move beyond descriptive analysis to further explore the complexities of experience over time [16]. LQR generates a rich source of data which has been used successfully for secondary analysis of data [11,17].

How analysis with this multidimensional data can be integrated is a particular challenge and is not well described or reported in the literature [4]. Papers tend to focus on either the cross-sectional or longitudinal (narrative) data. This means that the longitudinal aspects of the study, time and change, are often poorly captured. In particular the reporting of cross-sectional data alone can lead to descriptions of each time point rather than focusing on the changes between time points. Studies may have the explicit aim to focus on one or other aspect of analysis and this will achieve different analysis and reporting. The addition of a theoretical framework can help to guide researchers during analysis to move beyond description.

The purpose of this paper is to reflect on the strategies used in an LQR programme and highlight ethical, practical, theoretical and methodological issues that need to be considered and addressed from the outset of a study, giving researchers in the field some direction and raising the debate and discussion among researchers on ways to develop and carry out LQR projects.

## Methods

We have carried out over the past six years a large LQR programme of research about experiences of symptoms in cancer patients [18-25]. This included interviews with patients from eight cancer diagnostic groups (and their caregivers) from diagnosis to three, six and 12 months later. As researchers working for the first time with longitudinal qualitative data we developed our research design and analysis strategy iteratively throughout the project. We have a particular focus on cancer patients, so this paper will have particular relevance for researchers interested in chronic and life limiting conditions.

As we were completing the analysis and dissemination of this large programme of research we wished to reflect on our experience of a health services research LQR project. As members of the core research team we felt that we had developed a great deal of experience in the development and management of such a project. We felt that if we pooled our knowledge we could suggest some important lessons learned from our experience. The authors met at regular intervals to identify the key aspects of the researchers’ experience of conducting this LQR project that we considered were not well addressed within the current literature. Issues were identified through brainstorming sessions among the investigators and consideration of past formal discussions (recorded or not) during the project duration. A final complete list was presented and discussed in an open meeting with a group of qualitative researchers from a supportive care research team and further discussions took place. Common issues that are relevant to any qualitative research and for which there is significant literature where left out, and only issues that were closely linked with LQR remained in the list for further discussion. Alongide our experience and consultation with experienced qualitative researchers, we have also searched the literature to find out if there is any clear information on each issues/topic. Recommendations, thus, were both experience-based and literature based, although due to lack of or limited literature around some of the issues discussed, experience-based recommendations were more common. This paper was developed to give examples of how specific ethical and practical issues in the project were tackled so they might stimulate debate and discussion amongst LQR researchers.

## Findings

We present the results of our discussions and suggested solutions below and these are summarized in Table 1.

**Table 1:** Summary of themes and suggested solutions

**Key theme**	**Issues arising**	**Approach used/suggested solutions**
Ethical issues: participant	Recruitment shortly after significant diagnosis	Treating doctor assessed participant prior to approach by researcher.
		Approached participants sensitively in order to build trust and develop relationships over long term
	Blurring of boundaries as relationships develop	Agreed plans to manage participant initiated contact about e.g. their treatment or health status (researchers did not give advice but referred participant to relevant health professional)
	Potential for patients to become unwell or die during study	Written distress policy for participants and the research team in place
		Ongoing consent recorded over the life of the project
Ethical issues: researcher	Developing relationships over time	Prepared researchers to manage difficult topics and emotions during the interview, and how management might change as relationships deepen
	Closure of relationships	
		Developed a supportive network for researchers (e.g. debriefing sessions post interview)
	Confidentiality – and sharing data over large research teams	Written procedures for managing ad-hoc or informal contacts with participants.
		Developed clear data transfer and management plans
	Management of participant fatigue in interviews	Ensure as the interview schedule changes due to new emerging topics that it is not over burdensome. Find new ways to ask questions to avoid repetition (do not merely add more questions)
		Involvement of service users in study design
Recruitment and retention of participants	Some groups of patients had high levels of attrition due to natural history of disease	Checked health status of participants before contacting them prior to next interview to ensure this was done sensitively
		Careful thought should be given to heterogeneity of the sample. The time points at which data is collected may have to be managed differently for sub-groups
Time	At what time points should data be collected?	We made a pragmatic decision about this and time points were the same for all participants.
		It may be more relevant to identify time points by key transitions in the patient’s journey or by consideration of previous literature or informed by theory
	Time should be explicitly included in the interview – to include changing illness perceptions	Looking forwards and backwards in interviews moves away from linear notions of time
		Encourage reflexivity in the participant as well as the researcher
		Asking participants to reflect on their experience from the previous interview
Data collection and management of resources	Management of time and resources – when working with a large data set	Ensure adequate time is included in project plans for project management and communication with participants
	Funding for LQR	Work with the funding bodies to consider LQR
	Research focus and topic guide evolves over time	Flexibility, openness and responsiveness to the data and emerging analysis and interpretation is a key skill for the LQR researcher
		Ask for advice about how to manage this from an ethics committee
Analyzing data	LQR data sets are large and complex and can be analyzed in multiple ways from different perspectives	Ensure adequate time to analyze data between interviews – even if analysis is preliminary
		Consider analysis of data within each case and as comparison between cases
		Consider if and how subgroups should be analysed – is there a strong theoretical or practical reason why some groups should be analysed separately?
		Consider the contribution of a number of different analysis strategies to the data and their strengths and weaknesses
		Consider analysing data in a number of different ways, to add alternative understandings of longitudinal data

### Ethical issues: participant related

Patients with cancer may be vulnerable, with a high symptom burden and poor prognosis, but patients still value being able to contribute their views [10,26]. Longitudinal research with this patient group is important but some ethical issues are amplified by collecting in-depth data from the same participants over time. Particular issues have been identified as intrusion (into people’s lives), distortion (of experience due to repeated contact, personal involvement and closure of relationships) and dependency [4].

We wished to interview patients shortly after diagnosis, which is a critical point in the patient pathway. Sensitive recruitment of participants soon after a life changing diagnosis, such as cancer, is important in building relationships and establishing a long term commitment to a study. Although building relationships and developing trust is essential this adds complexity to the role of the researcher involved in longitudinal research. Both the researcher and the researched can be affected by their involvement over time [27]. We found that on occasion patients did contact the research team for advice or information relating to their diagnosis. It is important that a research team have plans in place to manage this sort of situation without detriment to the relationship with the participant. There was a clear written distress policy for interviews and participants were given information about local support in case they wanted this after the interview.

There was a significant risk in our research that patients would become too unwell to participate or die between interviews. We sought consent from participants to access medical records and were able to check the health status of participants prior to contacting the participants to make arrangements for the next interview to ensure this was done sensitively. Consent was an ongoing process and was given in writing prior to the first interview and consent was checked verbally prior to each subsequent interview and also during the interview if a participant became upset or was talking about a particularly sensitive issue. The participant would be reminded that the tape recorder could be switched off at any time and the interview could be terminated at any time. If upset the participant would be given time to recover before the researcher asked if it was acceptable to continue with the interview. These procedures were built into the study protocol and the application for ethical approval.

### Ethical issues: researcher related

Researchers too can be affected by their role [27]. Despite good training and support protocols for researchers qualitative research can be emotionally challenging [27]. Building a relationship over time, hearing about distressing situations and the impact that diagnosis can have on everyday life and relationships is hard. Information may be disclosed to the researcher that has not been discussed with anyone else; this builds a bond between those involved. Researchers may see participants deteriorate and die. The research team needs to build a supportive network and procedures to ensure that researchers are well supported in their role. In our study we used debriefing for very stressful events and researchers had regular supervision with the study team. Peer support within the research team also proved important on a day to day basis. It has been suggested that professional counseling is made available for researchers for whom debriefing is not sufficient support [27].

Staff retention may be an issue over time. There is a tension between the need to build relationships with participants in difficult circumstances and researcher burn out. It is ideal that one researcher builds a relationship with a participant over time but due to staff turnover or sickness this may not always be possible. Changes in staffing on LQR projects need to be well managed; the participant should be made aware that a different researcher will interview them and the researcher should read through previous transcripts so that participants feel there is some continuity and they do not have to repeat their story.

“Escaping the field” [4] or closure of relationships that have been built over time requires thought. Participants in our studies were prepared for the longitudinal element and the closure of the relationships. Study information was clear so participants knew that they were going to be interviewed 4 times over the year, and researchers prepared participants for the last interview: when ringing to arrange last interview participants were reminded that it was the final visit. At the end of the last interview we asked participants how they had found the process of being involved in research and had an informal “debriefing” session with them. If patients died whilst on the study a card would be sent on behalf of the research team to offer condolences.

It is important to ensure the confidentiality is maintained throughout the project as personal details, such as addresses, may be kept for longer than in studies with a single data collection point. Any ad hoc correspondence, phone messages or emails, for example, from participants to update researchers on their condition, should be handled in line with ethical approval requirements. As data is collected over time and experiences may be bound in particular circumstances and contexts ensuring that participants are not identifiable becomes more pertinent. The “blurred boundaries” for example taking your “emotional work” home with you [27] may also need special attention in LQR. Wray et al. [27] report, in their study, taking telephone calls from participants at home and ensuring women got evidence based care. These are complex, grey areas in LQR and it may become harder to separate, or manage ethically, empathy as a human being and a wish to help people who are suffering, with the role of a researcher when relationships deepen over time. These issues may have implications for the confidentiality of participants’ identities and data.

Data may have to be shared across large teams; this may mean that the core research team loses control of the data set and it is important to ensure that all team members are working to the ethical principles agreed with the relevant ethics committee. Large volumes of data may be generated from LQR and consideration should be given to how this data is archived and stored for the required length of time stipulated by the university, hospital or other regulatory body. LQR data is a valuable resource for archiving, data sharing and secondary data analysis, and may be a requirement of some funding bodies. To date this has been more common for large qualitative population data sets and is a specialist service offered by some Universities. The correct ethical approval, and participant consent to this, should be sought at the outset.

It is important to consider how researchers will deal with participant fatigue; within quantitative studies much thought is given to the burden of lengthy repeated questionnaires, the same consideration should be made for LQR, particularly as new topics of interest may emerge during the course of the study and it is tempting just to add a few more questions to the interview. Focusing on the purpose of the research, finding different ways to ask questions can avoid repetition and participants anticipating questions and giving the “right” response [28]. It is also wise to involve patients or service users in the design of the research and ongoing management to get the participants’ perspective of burden and balance research interest with participants’ well being.

### Recruitment and retention of participants

We were successful in the recruitment of participants to the study. Patients were identified by the clinical team at the research site and then approached by a member of the research team to give information about the study. Once participants were recruited to the study retention was satisfactory. Recruitment and retention are important in all longitudinal studies. In qualitative studies sufficient participants are required at the last time point to ensure data saturation particularly if any new themes become evident at this point. We also wished to interview carers and this created a significant number of interviews at follow-up. We eventually made the decision not to interview some carers at follow-up as data was saturated. This created some difficulty with carer participants who valued this ongoing opportunity to ventilate feelings. The oversampling at the beginning (in order to have an adequate number of subjects at the last interview) was not a successful technique and overstretched the researchers and the data collection process unnecessarily.

There were two groups of patients where attrition was particularly poor: lung cancer patients (where 18 were recruited and four finished the study) and brain cancer patients (where 11 started and only one patient completed the fourth interview). For both of these groups there was a significant drop off after the third time point at six months. These attrition rates were not unexpected and almost all of these participants withdrew because they were too unwell or had died; this type of attrition may be unavoidable in some patient groups. All breast and gynecology patients completed all four interviews. Hence, a more selective approach to over-recruitment at the beginning of a LQR project is advocated, basing such decision on the outlook of participants over the timeline of the project. In some LQR studies it might be appropriate to develop newsletters or a web site with news of the study for participants to sustain interest. Good researcher communication skills are required to develop trust and convey the importance of the project to participants in the initial stages of the project. We have field notes that suggest that participants found participation in the study beneficial and this may also have contributed to our successful retention rates in populations with better health and survival.

The attrition in the sample highlights the complexity of having a heterogeneous sample in longitudinal research. We were well aware at the outset of the different disease trajectories of the tumor groups but for the purposes of analysis we designed the data collection points to be the same for all patients. In retrospect this was not entirely appropriate as there were different disease and treatment trajectories within each diagnostic group. In future research we would think differently about timing of interviews and link it to, for example, critical incidents rather than having set time points. Careful thought should be given to heterogeneity of the sample; by sampling over a number of cancer diagnostic groups we complicated our analysis making it difficult to draw together the experiences of patients with different disease trajectories. It may have been a better strategy to sample for heterogeneity within, for example, patients with advanced cancer. While heterogeneity in qualitative research is a desirable sampling feature, in LQR it is the “change” in events that is of more importance, and depicting change in very heterogeneous populations may not be so meaningful. Hence, defining clearly what an appropriate sample is for a given LQR study and understanding the trajectory of this sample over time are highly important considerations.

### Time

Issues of time and timing are of importance. Longitudinal research often focuses on change: how does coping or experience change? or how do participants manage change over time? [1]. Quantitative longitudinal research, such as cohort studies, assumes linearity of experiences and that people may experience time in the same way. However, the notion of time in a disease trajectory is complex. The difference between clock time and embodied time (or the experience of time) of the cancer patient has been recently illustrated in lung cancer, and this research highlights the lack of relationship between these two conceptualizations of time [29]. The differences between research time and biographical time have been explored elsewhere too [1]. Thus, consideration needs to be given to how time is defined in the study by the participants and by the research team.

One of the central issues we faced in this study was about the nature of time. As discussed above we identified set time points for data collection at the outset. However, we discovered that it is important to balance the pragmatics of a research design with flexible notions of time. We had significant attrition after the data collection point at six months and in retrospect we had not factored in the short disease trajectories of some patients or that some patients may have different notions of time. It may have been more useful to identify potential turning points or defining moments, from initial interviews, previously published research or clinical understanding of disease and focus on those rather than identifying set time points. For example, we know that the end of treatment, be that palliative or curative, is a significant time for patients [30,31] but treatment duration may not fall neatly into the first three months after diagnosis. That said, the focus of interviews should not be about “concrete events, practices, relationships and transitions which can be measured in precise ways, but with the agency of individuals in crafting these processes [32], p 192.” However, defining moments do often lead to change, in experience, coping or relationships and are useful points to tap into participants’ experiences. However, on a practical level, it would have been very difficult with our large data set to keep track of these critical incidents for every participant and to be able to organize researcher appointments to conduct interviews.

Issues of time need to be explicitly placed within the interview, an aspect we could have strengthened in our study. Looking both forwards and backwards in time moves away from linear notions of time as discussed above, asking participants to reflect on the content of their previous interviews. One way of doing this may be to encourage participants to approach the interview with reflexivity [33], a concept we are familiar with as researchers but in longitudinal research may be as important for the participant. For example, an issue that seems important for participants in the short term may not prove to be as important in the long term with the benefit of hindsight or increased understanding of the context [34]. This tentative or provisional, often contradictory, understanding makes analysis complex. As researchers we must endeavour to understand these complexities and make sense of them.

McLeod [33] suggests that reflexivity within the interview did not work for all of her research participants (in a study of school children) and is a point worth pursuing as we further develop our understanding of this methodology with patients. Reflexivity on a health state is complex for patients and it has been suggested that interviewing the ill may pose particular difficulties for the researcher [35,36], [a]s sick people, participants are unfamiliar with their everyday worlds, and they are often incapable of describing their condition and perceptions, so that researchers have difficulty in obtaining data to comprehend, interpret and generally conduct their research. … When researching participants who are sick, these methodological problems result in decisions about the timing of data collection, challenges to validity and reliability, and debates about who should be conducting the research [35], p 538.

Longitudinal qualitative research may in some way solve some of these issues as researchers will have the chance to incorporate changing illness perceptions into data collection and analysis. Patients whose illness has a long term impact will develop vocabulary and a way of expressing their illness experience in a way that patients with an acute episode will not. These changing perceptions, often moving from a lay perspective to one of the patients managing and controlling their illness [37], needs to be factored into analysis.

### Data collection and management of resources

One of the main difficulties with LQR is the time and resources that are required to undertake a study. Dealing with a large data set can bring logistical challenges and there is a significant amount of time spent on project management, keeping up to date with participants, sending reminders and checking on a patient’s status. Analysis between interviews, across the participants and longitudinally within the individual narrative, can be a significant challenge in LQR.

There are no guidelines about how long a longitudinal study should be (although at least 2 points are necessary to examine change [3]) or how often data needs to be collected; this should be determined by the processes and population under investigation and the research question. Many health/patient related studies are short in duration, one to two years, in comparison to LQR in the social sciences where issues, such as transitions in identity from child to adult, are investigated over decades. This may of course be because of differences in the issues/processes under investigation but may also reflect research funding in health care which is often limited to a fixed duration. This poses problems for a research team who wish to follow a population for a number of years and requires ongoing generation of funds to complete the research.

The topic guide and the focus of the interview may change over time, this may prove challenging when seeking ethical approval for a study. Ethics committees usually ask for all documentation including topic guides prior to giving an opinion. Our interview schedule had broad questions both to comply with ethical approval procedures and to allow participants to talk about what is important for them at the time of each interview. Example opening questions include “How have you been feeling physically this past month” or “How have you been feeling emotionally this past month”. Developing a relationship with an ethics committee and seeking guidance about how to approach this with the committee is advisable.

LQR is a prospective approach and therefore can give a different perspective on processes. Issues that seem very important at one time point may change with the perspective of time and processes may change the way experiences are viewed. One off qualitative interviews rely on recall, for example, asking about symptom experience at diagnosis when a patient is several months away from that point. There will always be some element of retrospective discussion in an LQR interview but with a focus on change over time, this can be aided by summarizing or reflecting on the previous interview. As data is collected prospectively, causation, the temporality of cause and effect, and the processes or conditions by which this happens can also be explored in the data [4].

As we describe below, the richness of the interview content and overwhelming amount of data made it difficult to analyze in-depth each interview before the next one, an issue also been reported in other studies [27]. When this is the case we would propose that a preliminary analysis and summary of the interview is made so that the next interview can commence with a recap of what was previously discussed. Subsequent interviews could start by the interviewer providing a short summary of themes they have identified from the last interview and asking the participant to reflect on this summary of experiences before moving on to ask how the participant is feeling now and what has changed for them since the last interview. This more selective interview approach in subsequent interviews may also decrease the amount of data collected, easing the analysis and making the data collected more focused and less overwhelming for the researcher. Indeed we have noticed that often subsequent interviews tended to be shorter than the initial one. This helps the researcher and participant to keep the focus on longitudinal elements, what has changed since last time, why has this happened? Preliminary analysis will also highlight emerging themes to be further pursued in later interviews.

Using LQR researchers can respond to a change in focus and interviews can be adapted to the individual narratives. This is particularly useful as at the outset it is often not clear what the important processes are over time. Thus much data collected in the initial stages may not be relevant in the emerging processes over time, and data collection necessarily will become more focused at later time points. Flexibility and responsiveness to the data and emerging analysis and interpretation is a key skill for the LQR researcher.

### Analyzing data

Longitudinal qualitative data analysis is complex and time consuming. A longitudinal analysis occurs within each case and as comparison between cases. The focus is not on snapshots across time (a cross-sectional design will achieve this) but “to ground the interviews in an exploration of processes and changes which look both backwards and forwards in time [32], p194.”

Holland [4] synthesizes two approaches to analyzing data and suggests some questions to guide analysis. Firstly, framing questions focus on the contexts and conditions that influence changes over time, she gives the example, “what contextual and intervening conditions appear to influence and affect participant changes over time? [4].” Descriptive questions generate descriptive information about what kinds of changes occur, for example, “what increases or emerges through time? [4].” These two types of questions move the researcher forward to develop deeper levels of analysis and interpretation.

Data collection and analysis should be informed by the research question, data collection methods and theoretical perspective, if one is being used from the outset. It may be possible to anticipate whether cross-sectional or longitudinal analysis would be the most helpful method of answering the research question. Considering these issues at the outset may allow the researcher to be alert to themes in the data during analysis whilst keeping an open mind to emerging issues.

As described above we planned to analyze each interview before moving onto the next interview with each participant to allow reflexivity of the researcher and participant and to focus on “processes and changes” rather than snapshots. Due to the volume of data it was not always possible to do this and this is certainly a limitation of our work and may reflect the predominance of cross-sectional data in our reporting of the studies.

We decided to analyze each tumor group separately rather than across the whole sample as it was clear that there were significant differences in these populations due to different disease trajectories and symptom experience. There was a different analysis and theoretical perspective taken in each analysis reflecting that data from each tumor group. McLeod [33] suggests that the nature of longitudinal data means that multiple theoretical frameworks may be useful to analyses and interpretation and the use of different paradigms may lead to new insights and interpretations.

Interpretative Phenomenological Analysis was used in lung cancer analysis [21], Interpretative Description with lymphoma data [20], content or thematic analysis using Leventhal’s self-regulation theory, the theoretical framework for the study, was used for gynecological, brain, and head and neck cancer data analysis [18,22,23], and thematic narrative analysis for breast cancer patients, The above approach took into consideration the data analysis experience of the researchers involved or the type of information collected through the interviews. For example, the analysis of breast cancer patients’ accounts [25] lead itself to narrative analysis because the women expressed their feelings much more than other groups and we analysed the data through patient stories about their cancer journey; this fitted well with the approach to data generation and Frank’s [38] concept of the cancer journey was used as the theoretical lens though which data were analyzed. In data from other diagnostic groups the unit of analysis was often the whole interview, as in the case of patients with head and neck cancer, where coding units in the first interview were assessed for presence and information in subsequent interviews. This captured well some experiences over time, such as the continuous nature of fatigue and tiredness over time, or the attempts for maintaining normality which were evident only after T2, increasing in complexity at T3 and T4 [22]. Detailed practical examples are presented in the respective papers [18-25] and a summary of the themes alongside other qualitative research related to symptom experience of cancer patients is presented in a meta-synthesis of these data [39].

Our analyses have highlighted new insights into the symptom experiences of patients with cancer. Utilizing multiple analysis strategies and theoretical perspectives has its strengths and allows comparison and gives direction for reanalysis and further interpretation of this important research resource.

### Recommendations

Through reflecting on and describing our experiences we have identified broad recommendations for undertaking LQR projects in health research which we hope will stimulate debate amongst qualitative researchers.

• We would recommend incorporating a theoretical perspective (if appropriate to the methodology), that encompasses concepts such as time or the experience of change. This may help researchers keep the analysis “alive” to longitudinal aspects of analysis and move beyond descriptions of experience at each time point to explore change between time points.

• Qualitative researchers are familiar with complex ethical issues involved in being in the field. However, there are some ethical issues that are amplified whilst undertaking LQR, and require careful consideration and planning, such as how relationships are built and sustained over time whilst adhering to ethical practices, how relationships are ended, maintaining confidentiality over time and managing distress in participant and researchers.

• Good project management is essential when working with large data sets. Ensure adequate time is included in project plans for project management and communication with participants.

• Developing good team working is important; there are advantages to working with large teams which may be an unfamiliar way of working for qualitative researchers. Different perspectives can be brought to bear on the analysis making it richer and generating new insights. Communication is particularly important when analysis is undertaken by researchers who have not been involved in collecting data.

• We would encourage researchers to consider multiple methods of analysis and secondary analysis within the same data set to explore the rich data that is generated.

• We have clearly identified that longitudinal research with patients with a poor prognosis and experiencing long term challenges is worthwhile. However, thought needs to be given to the timing of data collection and the heterogeneity of the sample. Support for participants and researchers, and any additional ethical considerations, should be built into protocols as there is an increased burden for all involved in LQR.

• We recommend that from the outset the research team should consider how the volume of data can be managed and consider practical issues such as timing of interviews so data can be transcribed and analyzed in time for the next round of interviews. This early analysis may help keep the focus on change and transitions rather than description of events.

•Funders of research may be unfamiliar to funding longitudinal qualitative research and recommend that a strong case for the added value of this method should be made.

## Discussion

This paper has explored our experience of LQR and highlighted areas where we have learned a great deal about the methodology. During this longitudinal project we developed expertise in managing practical and ethical issues, tried different analysis strategies to look for alternative ways of examining data and understanding the experience of participants. There have been successes in the strategies we have used and areas in retrospect that we could have worked differently. For example, ensuring sensitivity during initial recruitment and subsequent contacts, putting procedures in place from the outset of the study to manage issues such as patient distress during interviews and patient initiated contact regarding health issues during data collection all helped the researchers to build trusting relationships with participants. These factors, together with researcher continuity, were important in helping to maintain good recruitment rates for participants with better health and survival rates throughout the study.

It is important to note that findings were generated from one particular study and issues highlighted here reflect the conduct of this study. There are other methodological issues that may be illustrated better through other examples of LQR research and we would encourage researchers to publish methodological issues highlighted by their studies to strengthen debate in this area. Although we consider that there are general lessons to be learned from our experience, which can be usefully considered by other researchers, we acknowledge that there may be aspects of the study, particularly the heath status of the participants that will not necessarily be broadly relevant. For this reason we do consider that this paper will have particular relevance for researchers interested in chronic and life limiting conditions.

We found that when seeking guidance for the project published literature was limited in highlighting debates about LQR focusing on the reporting of findings rather than developing debate about this emerging methodology. Much of the methodological literature cited in this paper comes from the social science literature where there is a long standing tradition of LQR and where debates about LQR with schoolchildren or other healthy populations in society are well rehearsed. There is little literature that examines the methodology in the context of health services research and whether there are particular issues about following participants through the trajectory of their illness to recovery, living with impairments or death. This paper has started to highlight some of the areas where further methodological exploration would be valuable.

One of the ongoing debates in qualitative methodology is how quality and credibility are evaluated [40,41]. There is little debate about whether LQR poses additional questions about quality. We have highlighted where, for example, there may be heightened concerns about ethical conduct, and using multiple methods of analysis. Longitudinal analysis is complex and is often reported a-theoretically and descriptively [13-15] and this also has implications for the quality and credibility of LQR. It may be that established guidance for the evaluation of qualitative research can be utilised with LQR but little exploration of this can be found in the published literature. Summaries of the researcher’s interpretation of a data collected in a previous interview when discussed with participants at a subsequent interview can enhance the credibility of the data. We have highlighted some ways in which these aspects of LQR can be enhanced, and by providing a record of our experiences it can help to start standardising a process by which QLR can be conducted which can enhance the credibility of research and quality of data collected.

LQR is an increasingly utilised methodology in health services research, for example in the development and evaluation of complex health interventions or to study transitions in recovery or long term illness. The findings presented in this paper are important as they begin to identify areas of LQR where there is potential for debate and multiple perspectives on these would be valuable.

Additional research and inquiry is also essential to further develop the methodology. There is little published work about rigour in LQR, and it would be worth investigating whether additional elements should be added to accepted conceptualizations of the quality of qualitative research so judgments can be made about the rigour of research. Research to explore participants’ perspectives of being in a longitudinal study would be valuable as there may be additional burden to the participant, emotional and practical, of being involved in LQR. Eliciting participants’ insights into their experiences of participation may give us greater insight into the method itself.

## Conclusions

This paper has highlighted specific methodological, practical and ethical issues identified in an LQR programme of research about experiences of symptoms in cancer patients in the first year after diagnosis. The study itself has highlighted useful insights into these experiences and allowed examination of data from multiple perspectives, but importantly has been an important learning opportunity of the research team. Next steps may include agreement among the qualitative research community about standardization of the process, identification of LQR research questions that would be distinct from what can be achieved from cross-sectional work, and influencing funders for the value and uniqueness of this methodological approach.

## Competing interests

The authors declared no conflicts of interest with respect to the authorship and/or publication of this article.

## Authors' contributions

Conception of paper: AM, LC. Acquisition of original data: AM, LB. Interpretation of data: All authors. Drafting paper: LC. Critical revisions: AM, LB. Final approval: all authors.

## Pre-publication history

The pre-publication history for this paper can be accessed here:

http://www.biomedcentral.com/1471-2288/13/14/prepub

## References

[B1] Thomson R Plumridge L Holland J Editorial Int J Soc Res Methodol 2003 6 3 185 187 10.1080/1364557032000091789

[B2] Maher J McConnell H New pathways of care for cancer survivors: adding the numbers Br J Cancer 2011 105 S1 S5 S10 22048033 10.1038/bjc.2011.417 PMC3251951

[B3] Saldana J Longitudinal Qualitative Research: Analyzing Change Through Time 2003 Walnut Creek, CA: AltaMira Press

[B4] Holland J Qualitative Longitudinal Research: Exploring ways of researching lives through time. Real Life Methods Node of the ESRC National Centre for Research Methods Workshop held at London South Bank University 2007 Retrieved from http://www.reallifemethods.ac.uk/training/workshops/qual-long/documents/ql-workshop-holland.pdf

[B5] Holland J Thomson R Henderson S Qualitative longitudinal research: A discussion paper, Working Paper No. 21, Families & Social Capital ESRC Research Group 2006 London South Bank University http://www.lsbu.ac.uk/ahs/downloads/families/familieswp21.pdf

[B6] Oakley A Strange V Bonell C Allen E Stephenson J and the RIPPLE Study Team Process Evaluation in Randomised Controlled Trials of Complex Interventions Br Med J 2006 332 413 416 10.1136/bmj.332.7538.413 16484270 PMC1370978

[B7] Yedidia MJ MacGregor B Confronting the Prospect of Dying: Reports of Terminally Ill Patients J Pain Symptom Manage 2001 22 4 807 819 10.1016/S0885-3924(01)00325-6 11576797

[B8] Kendall M Murray SA Tales of the Unexpected: Patients Poetic Accounts of the Journey to a Diagnosis of Lung Cancer: A Prospective Serial Qualitative Interview Study Qualit Inq 2005 11 5 733 751 10.1177/1077800405276819

[B9] Axelsson L Randers I Jacobson SH Klang B Living with haemodialysis when nearing end of life Scand J Caring Sci 2012 26 45 52 10.1111/j.1471-6712.2011.00902.x 21605154

[B10] Murray SA Kendal M Carduff E Worth A Harris F Lloyd A Cavers D Grant L Sheikh A Use of serial qualitative interviews to understand patients evolving experiences and needs Br Med J 2009 339 b3702 10.1136/bmj.b3702 19786485

[B11] Murray SA Marilyn K Boyd K Grant L Gill H Sheikh A Archetypal trajectories of social, psychological, and spiritual wellbeing and distress in family care givers of patients with lung cancer: secondary analysis of serial qualitative interviews Br Med J 2010 340 c2581 10.1136/bmj.c2581 20538635 PMC2883691

[B12] Krishnasamy M Wells M Wilkie E Patients and carer experiences of care provision after a diagnosis of lung cancer in Scotland Support Care Cancer 2007 15 3 327 332 10.1007/s00520-006-0129-3 16951952

[B13] Taylor C Richardson A Cowley S Surviving cancer treatment: An investigation of the experience of fear about, and monitoring for, recurrence in patients following treatment for colorectal cancer Eur J Oncol Nurs 2011 15 3 243 249 21530395 10.1016/j.ejon.2011.03.010

[B14] Kennedy F Harcourt D Rumsey N The shifting nature of women’s experiences and perceptions of ductal carcinoma in situ J Adv Nurs 2012 68 856 867 10.1111/j.1365-2648.2011.05788.x 21790736

[B15] McCaughan E Prue G Parahoo K McIlfatrick S McKenna H Exploring and comparing the experience and coping behaviour of men and women with colorectal cancer after chemotherapy treatment: a qualitative longitudinal study Psycho-Oncol 2012 21 64 71 10.1002/pon.1871 21132680

[B16] McCann L Illingworth N Wengstrom Y Hubbard G Kearney N Transitional experiences of women with breast cancer within the first year following diagnosis J Clin Nurs 2010 19 13–14 1969 1976 20936753 10.1111/j.1365-2702.2009.03134.x

[B17] Murray SA Kendall M Grant E Boyd K Barclay S Sheikh A Patterns of Social, Psychological, and Spiritual Decline Toward the End of Life in Lung Cancer and Heart Failure J Pain Symptom Manage 2007 34 4 393 402 10.1016/j.jpainsymman.2006.12.009 17616334

[B18] Lopez V Copp G Brunton L Molassiotis A Symptom Experience in Patients with Gynecological Cancers: The Development of Symptom Clusters through Patient Narratives J Support Oncol 2011 9 64 71 10.1016/j.suponc.2011.01.005 21542413

[B19] Lopez V Copp G Molassiotis A Male Caregivers of Patients with Breast and Gynecologic Cancer: Experiences From Caring for Their Spouses and Partners Cancer Nurs 2012 35 402 410 10.1097/NCC.0b013e318231daf0 22067685

[B20] Johansson E Wilson B Brunton L Tishelman C Molassiotis A Symptoms Before, During, and 14 Months After the Beginning of Treatment as Perceived by Patients With Lymphoma Oncol Nurs Forum 2010 37 E105 E113 10.1188/10.ONF.E105-E113 20189909

[B21] Lowe M Molassiotis A A longitudinal qualitative analysis of the factors that influence patient distress within the lung cancer population Lung Cancer 2011 74 344 348 10.1016/j.lungcan.2011.03.011 21511356

[B22] Molassiotis A Rogers M Symptom experience and regaining normality in the first year following a diagnosis of head and neck cancer: a qualitative longitudinal study Palliat Support Care 2012 10 197 204 10.1017/S147895151200020X 22613011

[B23] Molassiotis A Wilson B Brunton L Chaudhary H Gattamaneni R McBain C Symptom experience in patients with primary brain tumours: A longitudinal exploratory study Eur J Oncol Nurs 2010 14 410 416 10.1016/j.ejon.2010.03.001 20363189

[B24] Stamataki Z Burden S Molassiotis A Weight Changes in Oncology Patients During the First Year After Diagnosis: A Qualitative Investigation of the Patients’ Experiences Cancer Nurs 2011 34 401 409 10.1097/NCC.0b013e318208f2ca 21252641

[B25] Tighe M Molassiotis A Morris J Richardson J Coping, meaning and symptom experience: A narrative approach to the overwhelming impacts of breast cancer in the first year following diagnosis Eur J Oncol Nurs 2011 15 226 232 10.1016/j.ejon.2011.03.004 21511530

[B26] Terry W Olson L Ravenscroft P Wilss L Boulton-Lewis G Hospice patients views on research in palliative care Int Med J 2006 36 7 406 413 10.1111/j.1445-5994.2006.01078.x 16780445

[B27] Wray N Markovic M Manderson L Researcher Saturation: The Impact of Data Triangulation and Intensive-Research Practices on the Researcher and Qualitative Research Process Qual Health Res 2007 17 10 1392 1402 10.1177/1049732307308308 18000078

[B28] Farrall S What is qualitative longitudinal research? Papers in Social Research Methods Qualitative Series, Paper 11. LSE Methodology Institute 2006 http://www2.lse.ac.uk/methodologyInstitute/pdf/QualPapers/Stephen-Farrall-Qual%20Longitudinal%20Res.pdf

[B29] Lövgren M Hamberg K Tishelman C Clock time and embodied time experienced by patients with inoperable lung cancer Cancer Nurs 2010 33 1 55 63 10.1097/NCC.0b013e3181b382ae 19926976

[B30] Armes J Crowe M Colbourne L Morgan H Murrells T Oakley C Palmer N Young A Richardson A Patients’ Supportive Care Needs Beyond the End of Cancer Treatment: A Prospective, Longitudinal Survey J Clin Oncol 2009 27 6172 6179 10.1200/JCO.2009.22.5151 19884548

[B31] Ganz PA Kwan L Stanton AL Krupnick JL Rowland JH Meyerowitz BE Bower JE Belin TR Quality of Life at the End of Primary Treatment of Breast Cancer: First Results From the Moving Beyond Cancer Randomized Trial J Nat Cancer Inst 2004 96 5 376 387 10.1093/jnci/djh060 14996859

[B32] Neale B Flowerdew J Time, texture and childhood: The contours of longitudinal qualitative research Int J Soc Res Methodol 2003 6 3 189 199 10.1080/1364557032000091798

[B33] Mcleod J Why we interview now–reflexivity and perspective in a longitudinal study Int J Soc Res Methodol 2003 6 3 201 211 10.1080/1364557032000091806

[B34] Plumridge L Thomson R Longitudinal qualitative studies and the reflexive self Int J Soc Res Methodol 2003 6 3 213 222 10.1080/1364557032000091815

[B35] Morse J Researching Illness and Injury: Methodological Considerations Qual Health Res 2000 10 4 538 546 10.1177/104973200129118624 11010077

[B36] Morse J Gubrium J, Holstein J Interviewing the Ill Handbook of Qualitative Interviewing 2002 Thousand Oaks: Sage 317 328

[B37] Calman L Patients’ views of nurses’ competence Nurse Educ Today 2006 26 719 725 10.1016/j.nedt.2006.07.016 17014931

[B38] Frank A The Wounded Storyteller: Body, Illness and Ethics 1995 Chicago: University of Chicago Press

[B39] Bennion AE Molassiotis A Qualitative research into the symptom experiences of adult cancer patients after treatments: a systematic review and meta-synthesis Support Care Cancer 2013 21 9 25 10.1007/s00520-012-1573-x 22972487

[B40] Cohen D Crabtree B Evaluative Criteria for Qualitative Research in Health Care: Controversies and Recommendations Ann Fam Med 2008 6 4 331 339 10.1370/afm.818 18626033 PMC2478498

[B41] Devers KJ How will we know “good” qualitative research when we see it? Beginning the dialogue in health services research Health Serv Res 1999 34 5 Pt 2 1153 88 10591278 PMC1089058

